# The impact of percutaneous endoscopic gastrostomy on nutritional status and survival in cervical esophageal cancer patients undergoing chemoradiotherapy

**DOI:** 10.3389/fnut.2025.1521239

**Published:** 2025-06-25

**Authors:** Zijun Wen, Xing Liu, Yingqi Zhong, Haier Zhou, Guoming Xiao, Zhongying Huang, Lihui Chen

**Affiliations:** State Key Laboratory of Oncology in South China, Guangdong Provincial Clinical Research Center for Cancer, Sun Yat-sen University Cancer Center, Guangzhou, China

**Keywords:** cervical esophageal squamous cell carcinoma, percutaneous endoscopic gastrostomy, CONUT score, body mass index, overall survival

## Abstract

**Objective:**

This retrospective study aimed to study the effects of percutaneous endoscopic gastrostomy (PEG) on nutritional status and overall survival (OS) of cervical esophageal cancer (CEC) patients who received concurrent chemoradiotherapy.

**Methods:**

Thirty-four CEC patients who underwent concurrent chemoradiotherapy (CCRT) were retrospectively reviewed. A series of nutritional indicators: controlling nutritional status (CONUT) score, weight, body mass index, albumin, lymphocyte counts, hemoglobin (HGB) was introduced to evaluate the nutritional status between patients with or without PEG.

**Results:**

Among the 34 patients, 18 received PEG placement (PEG group) and 16 did not (Non-PEG group). The median survival for the PEG group was 38.0 months (range, 6.0–60.3), and for the Non-PEG group, it was 43.5 months (range, 21.5–162.8). The 2, 3, and 4 year OS rates for the PEG group were 81.9% (95% confidence interval [CI]: 63.2–100%), 54.3% (95% CI: 25.5–83.1%), and 32.6% (95% CI: 0.0–63.6%), respectively, while the Non-PEG group had 2, 3, and 4 year OS rates of 100% (95% CI: 83.0–100%), 82.1% (95% CI: 59.2–100%), and 49.2% (95% CI: 11.4–87.0%), respectively. There was no significant difference in the OS between the PEG group and the Non-PEG group (*p* = 0.095, hazard ratio [HR] 0.398, 95% [CI] 0.135–1.173). In the nutritional index, changes in HGB were significantly correlated with PEG (*p* = 0.016). Multivariate analysis results showed: weight loss ≥5% (*p* = 0.041, HR = 5.664, 95% CI: 1.075–29.846) and a CONUT score ≥4 (*p* = 0.01, HR = 15.223, 95% CI: 1.935–119.783) were independent prognostic factors for OS.

**Conclusions:**

Weight loss during chemoradiotherapy and higher CONUT scores may decrease the OS rate for CEC patients. However, PEG insertion did not affect the OS rate.

## Introduction

Esophageal cancer (EC) ranks as the eighth most prevalent malignancy globally. China exhibits notably high incidence rates of esophageal cancer within East Asia and worldwide, coupled with one of the highest mortality rates for this disease (1). Cervical esophageal cancer (CEC) comprises 2–10% of all cases of EC (2). The predominant histological type of CEC in Asia is squamous cell carcinoma (SCC) (3). Cervical esophageal squamous cell carcinoma (CESCC) commonly extends upward to the hypopharynx or downward to the thoracic esophagus (4).

CEC patients often experience progressively worsening dysphagia, initially manifesting as difficulty swallowing solid foods, which eventually extends to soft foods and, ultimately, affects the swallowing of liquids and saliva (5). Concurrent chemoradiotherapy is recommended as the standard treatment for CESCC according to the National Comprehensive Cancer Network (NCCN) guidelines. Radiation therapy can induce mucosal inflammation, dysphagia, and stricture in the affected areas. This progression may lead to escalating pain and difficulty swallowing, significantly reducing oral food intake (6).

Nutritional disorders are highly prevalent in patients with EC (7, 8). Malnutrition is defined as a condition resulting from inadequate intake or absorption of nutrients, leading to alterations in body composition, including reduced fat-free mass and muscle mass. This condition impairs both physical and mental functions and worsens clinical outcomes. It can compromise immune function, performance status, muscle function, quality of life, response to chemotherapy, and overall survival (OS) (9, 10). Studies have shown that malnutrition is associated with poor clinical outcomes in patients with EC (11).

Percutaneous endoscopic gastrostomy (PEG) tube placement can reduce nutritional risk in patients with inadequate nutritional intake. Since its introduction by Gauderer and Ponsky in 1980 for pediatric patients with inadequate nutritional intake, PEG has transformed the practice of feeding tube placement (12). It offers a safe and reliable alternative to gastrostomy, avoiding laparotomy and carrying a low morbidity risk. With over 200,000 procedures performed annually in the United States, PEG has gained broad acceptance (13). Numerous studies have indicated that PEG feeding helps preserve body weight, improve quality of life, and reduce treatment interruptions (14–16).

Current research indicates that PEG is both safe and effective for providing nutritional support in patients with EC without harming the stomach or the esophagogastric junction (17). It is recommended that PEG feeding be considered the standard method for providing definitive nutritional support in patients with upper EC (18). However, concerns remain about early PEG tube insertion, including local wound infection, inflammation, abdominal pain, and feeding tube dependency (19–21). Several retrospective studies have evaluated the benefits of early PEG tube placement in EC patients undergoing multimodal treatments like chemoradiotherapy. While some patients with PEG tubes experienced improved nutritional management, most showed comparable outcomes in terms of perioperative complications, tolerance to chemoradiotherapy, and OS (22, 23).

Limited evidence exists on nutritional status changes in CESCC patients receiving PEG during definitive chemoradiotherapy. Further studies should assess PEG's nutritional efficacy and survival outcomes. This study examined whether PEG reduces treatment-related malnutrition and improves survival in CESCC patients.

## Materials and methods

### Patients

Between 22 February 2019 and 18 July 2022, 34 patients who attended Sun Yat-sen University Cancer Center (SYSUCC) were retrospectively analyzed. The study was approved by the independent ethics committee of SYSUCC and adhered to the ethical standards of the Declaration of Helsinki. All patients were pathologically diagnosed with CESCC and received treatment. Data were retrieved from the Hospital Information System and Electronic Medical Records at SYSUCC.

All CESCC patients were recommended to insert PEG before concurrent chemoradiotherapy (CCRT). However, some patients refused to insert PEG because they could still take liquid food orally. So, patients were stratified into two cohorts: the PEG group (*n* = 18) receiving prophylactic PEG tube placement before CCRT, and the Non-PEG group (*n* = 16) who declined prophylactic PEG placement. Patients in the PEG group underwent gastroscopy and contrast-enhanced abdominal CT to exclude contraindications (e.g., coagulopathy, anatomical anomalies), with informed consent obtained prior to the procedures.

### The inclusion criteria for this study were defined as follows

The inclusion criteria: primary tumor located in the cervical esophagus, histologically confirmed SCC, receipt of definitive CCRT, absence of distant metastasis, age of participants not exceeding 80 years.

### Ineligible criteria are as follows

The ineligible criteria: non-squamous cell histology or presence of multiple primary cancers, presence of distant metastases, history of malignant neoplasm, severe dysfunction of organs such as heart, liver, and kidney, patients who did not receive CCRT.

Using a specifically developed data entry form, we analyzed the characteristics of 34 patients. Key parameters included age at diagnosis, gender, body mass index, underlying diseases, history of tobacco and alcohol use, radiotherapy, chemotherapy, tumor staging, and use of PEG.

### Calculation of nutritional status

Nutritional parameters, including body weight, BMI, hemoglobin (HGB), serum albumin, lymphocyte count, total cholesterol, and CONUT score, were assessed longitudinally at six time points: baseline (diagnosis), weeks 1–3, the final week of CCRT, and 1 month post-CCRT. Temporal changes in these markers were compared between the PEG and Non-PEG groups. BMI was analyzed both as continuous values and as categorical classifications (normal range: 18.5–24.9 kg/m^2^ according to WHO criteria). Weight loss was calculated using the equation: (current weight – usual weight) / usual weight × 100%, and categorized into three groups: ≤ 5%, 5–10%, and >10% weight loss (24). The CONUT score was calculated by adding the scores of the following parameters: serum albumin level [≥3.5 g/dL (0 points), 3.0–3.4 g/dL (2 points), 2.5–2.9 g/dL (4 points), or < 2.5 g/dL (6 points)], total lymphocyte count [≥1,600 cells/μL (0 points), 1,200–1,599 cells/μL (1 point), 800–1,199 cells/μL (2 points), or < 800 cells/μL (3 points)], and total cholesterol level [≥180 mg/dL (0 point), 140–179 mg/dL (1 point), 100–139 mg/dL (2 points), or < 100 mg/dL (3 points)] (25). CONUT scores were categorized into three groups: 0–1 (good nutrition), 2–3 (fair nutrition), and ≥4 (poor nutrition) (26).

### Statistical analysis

OS was defined as the time from diagnosis to death from any cause, with surviving patients censored at the last follow-up. Survival curves were estimated using the Kaplan-Meier method and compared via the log-rank test. Cox proportional hazards regression was used for univariate and multivariate analyses. Continuous variables were expressed as median (IQR) and compared with the Mann-Whitney U test, as normality (assessed by Shapiro-Wilk test) was violated in most parameters. Categorical variables were analyzed using Fisher's exact test for small samples or Pearson's χ^2^ test with Yates' correction. Variables with *p* < 0.1 in univariate Cox regression (reported with HR, 95% CI, and exact *p-*values) were entered into the multivariate model. Multicollinearity was assessed using variance inflation factors (VIF >5 excluded). The proportional hazards assumption was verified via Schoenfeld residual tests; variables violating the assumption were modeled with time-dependent coefficients. A two-sided *p-*value < 0.05 after FDR correction was considered significant. Analyses were conducted in R 4.3.1.

## Results

### Patient characteristics

**Figure 1:**
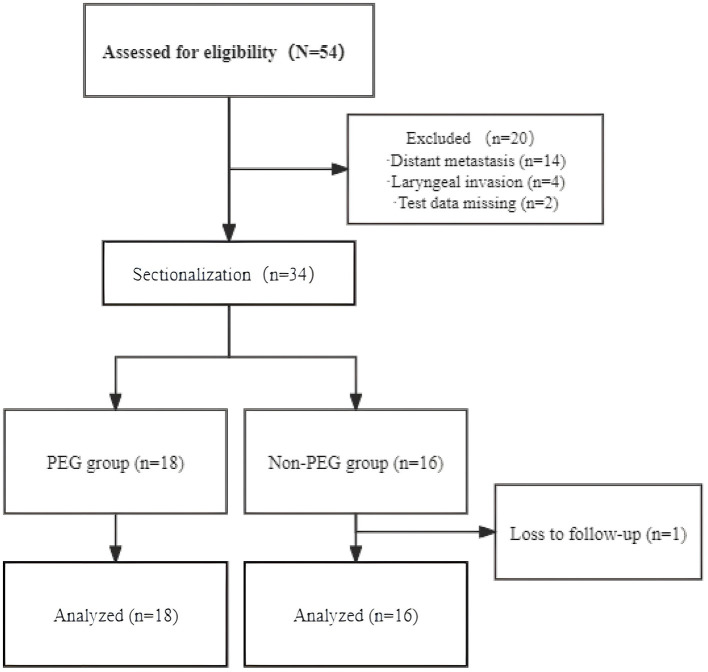
Schematic representation of eligible patients.

Thirty-four CEC patients were included in the final analysis and stratified into the PEG group (*n* = 18) and the Non-PEG group (*n* = 16) (Figure 1). The cohort consisted of 22 males (64.7%, 22/34) and 12 females (35.3%, 12/34), with a median age of 62 years (range: 30–73). Tumor staging revealed stage II in 4 (11.8%), stage III in 13 (38.2%), and stage IV in 17 (50.0%). Advanced-stage tumors (III/IV) were present in 30/34 (88.2%), with comparable proportions between the PEG (15/18, 83.3%) and Non-PEG (15/16, 93.8%) groups (*p* = 0.796, Fisher's exact test) (Table 1).

**Table 1:** Baseline demographics and disease characteristics.

**Characteristic**	**No. (%)**			
	**Overall (*n =* 34)**	**PEG group (*n =* 18)**	**Non-PEG group (*n =* 16)**	***p-*value**
Age, median (range), years	62 (30–73)	60.5 (30–73)	62 (46–73)	0.518
**Gender**				
Man	22 (64.7%)	13 (72.2%)	9 (56.3%)	
Woman	12 (35.3%)	5 (27.8%)	7 (43.8%)	0.331
**Smoker**				
No	22 (64.7%)	11 (61.1%)	11 (68.8%)	
Ever	12 (35.3%)	7 (38.9%)	5 (31.3%)	0.642
**Drinking history**				
No	26 (76.5%)	13 (72.2%)	13 (81.3%)	
Ever	8 (23.5%)	5 (27.8%)	3 (18.8%)	0.536
**Comorbidities**				
No comorbidity	23 (67.6%)	12 (66.7%)	11 (68.8%)	
Hypertension	7 (20.6%)	3 (16.7%)	4 (25%)	
Diabetics	4 (11.8%)	3 (16.7%)	1 (6.3%)	0.585
**Clinical T-stage**				
T1, T2	8 (23.5%)	5 (27.8%)	3 (18.8%)	
T3	11 (32.4%)	2 (11.1%)	9 (56.3%)	
T4	15 (44.1%)	11 (61.1%)	4 (25%)	0.120
**Clinical N-stage**				
N0	7 (20.6%)	5 (27.8%)	2 (12.5%)	
N1	14 (41.2%)	10 (55.6%)	4 (25%)	
N2	8 (23.5%)	2 (11.1%)	6 (37.5%)	
N3	5 (14.7%)	1 (5.6%)	4 (25%)	0.056
**Pathohistological grading**				
GX	12 (35.3%)	5 (27.8%)	7 (43.8%)	
G1	1 (2.9%)	1 (5.6%)	0 (0%)	
G2	20 (58.8%)	12 (66.7%)	8 (50%)	
G3	1 (2.9%)	0 (0%)	1 (6.3%)	0.884
**AJCC overall clinical disease stage**				
I	0 (0%)	0 (0%)	0 (0%)	
II	4 (11.8%)	3 (16.7%)	1 (6.3%)	
III	13 (38.2%)	5 (27.8%)	8 (50%)	
IVA/IVB	17 (50%)	10 (55.6%)	7 (43.8%)	0.796
**Induction Chemotherapy**				
Yes	23 (67.6%)	11 (61.1%)	12 (75%)	
No	11 (32.4%)	7 (38.9%)	4 (25%)	0.388
**Radiation dose, Gy**				
Median (IQR)	60 (56–60)	60 (53–60)	60 (59–63)	0.195
**Radiotherapy frequency**				
Median (IQR)	28 (27-29)	28 (24-29)	28 (28-30)	0.102

PEG, percutaneous endoscopic gastrostomy; AJCC, American Joint Committee on Cancer, *p* < 0.05 was considered statistically significant.

All patients received definitive CCRT. The median radiation dose for the entire cohort was 60 Gy (IQR: 56–60), with no significant difference observed between the PEG group (60 Gy, IQR: 53–60) and the Non-PEG group (60 Gy, IQR: 59–63) (*p* = 0.195).

### Nutritional status and hematologic examination

Following CCRT, the mean weight change was 0.4 ± 2.75 kg in the PEG group and 0.2 ± 3.46 kg in the Non-PEG group (*p* = 0.46). The proportion of patients with BMI < 18.5 kg/m^2^ decreased from 5/18 (27.3%) to 3/18 (16.7%) in the PEG group, whereas the Non-PEG group exhibited an increase from 2/16 (12.5%) to 4/16 (25.0%) (Table 2).

**Table 2:** Nutrition-Related characteristics: comparison between PEG group and Non-PEG groups.

**Parameter**	**No. (%)**			
	**All patients (*n =* 34)**	**PEG group (*n =* 18)**	**Non-PEG group (*n =* 16)**	***p*-value**
**Body weight change (kg)**				
Mean ± SD	0.25 ± 0	0.4 ± 2.75	0.2 ± 3.46	0.460
**Percentage of weight change (%)**				
Mean ± SD	0.47 ± 0.05%	0.84 ± 0.05%	0.38 ± 0.05%	0.437
**Weight loss% class during CCRT**				
< 5%	27 (79.41%)	15 (83.33%)	13 (81.25%)	
5–10%	4 (11.76%)	3 (16.67%)	1 (6.25%)	
>10%	2 (5.88%)	0 (0%)	2 (12.50%)	0.965
**BMI category at start of treatment (kg/m** ^2^ **)**				
< 18.5	7 (20.59%)	5 (27.78%)	2 (12.50%)	
18.5–24.99	22 (64.70%)	10 (55.56%)	12 (75.00%)	
25–29.99	4 (11.76%)	3 (16.67%)	1 (6.25%)	
≥30	1 (2.94%)	0 (0%)	1 (6.25%)	0.904
**BMI category at treatment completion (kg/m2)**				
< 18.5	7 (20.59%)	3 (16.67%)	4 (25.00%)	
18.5–24.99	23 (67.65%)	13 (72.22%)	10 (62.50%)	
25–29.99	4 (11.76%)	2 (11.11%)	2 (12.50%)	
≥30	0 (0%)	0 (0%)	0 (0%)	0.984
**laboratory parameters change, median (IQR)**				
Hemoglobin (g/L)	−4 (−52 to 45)	−4.5 (−28 to 45)	−5.5 (−52 to 20)	0.290
Albumin (g/L)	1.15 (−9.6 to 15.8)	1.4 (−8.6 to 15.8)	0.95 (−9.6 to 12.4)	0.470
Lymphocyte count (109/L)	−0.53 (−1.86 to 0.84)	−0.53 (−1.78 to 0.84)	−0.44 (−1.86 to 0.68)	0.452
Total cholesterol (mg/L)	0.53 (−1.25 to 2.3)	0.47 (−0.99 to 2.3)	0.66 (−1.25 to 1.82)	0.957
**CONUT score at start of treatment**				
0–1 (good)	19 (55.88%)	10 (55.56%)	9 (56.25%)	
2–3 (fair)	12 (35.29%)	6 (33.33%)	6 (37.50%)	
≥4 (poor)	3 (8.82%)	2 (11.11%)	1 (6.25%)	0.874
**CONUT score at end of treatment**				
0–1 (good)	7 (20.59%)	6 (33.33%)	1 (6.25%)	
2–3 (fair)	19 (55.88%)	6 (33.33%)	13 (81.25%)	
≥4 (poor)	8 (23.53%)	6 (33.33%)	2 (12.50%)	**0.018**

CCRT, concurrent chemoradiotherapy; BMI, body mass index; IQR, interquartile range; CONUT, controlling nutritional status.

No significant differences were observed in pre- and post-chemoradiotherapy laboratory parameters between the two groups, including HGB (*p* = 0.290), albumin (*p* = 0.470), lymphocyte count (*p* = 0.452), and cholesterol levels (*p* = 0.957).

At baseline, CONUT scores of 2–3 were observed in 6/18 (33.3%) PEG patients and 6/16 (37.5%) Non-PEG patients, while scores ≥4 were present in 2/18 (11.1%) and 1/16 (6.3%), respectively. Post-CCRT, the proportion of patients with scores of 2–3 remained unchanged in the PEG group (6/18, 33.3%) but significantly increased to 13/16 (81.3%) in the Non-PEG group (*p* = 0.018). For scores ≥4, post-treatment proportions were 6/18 (33.3%) in the PEG group and 2/16 (12.5%) in the Non-PEG group.

### Effects of PEG on nutritional status related indicators

**Figure 2:**
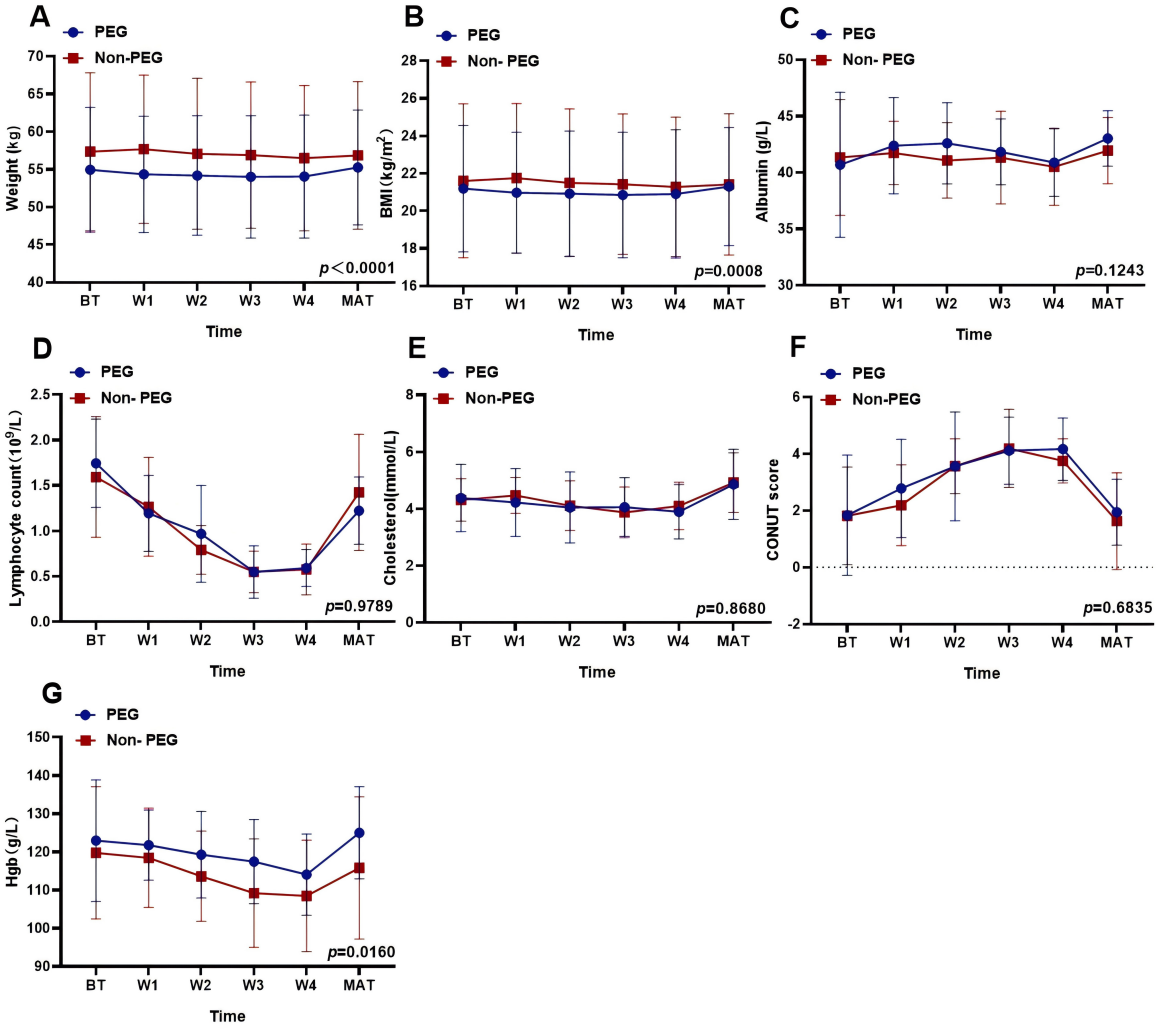
Effects of PEG on nutritional status related indicators. The effect of PEG on different nutritional indices: **(A)** Weight: *p* < 0.0001. **(B)** BMI: *p* = 0.008. **(C)** Albumin: *p* = 0.1243. **(D)** Lymphocyte counts: *p* = 0.9789. **(E)** Cholesterol: *p* = 0.8680. **(F)** CONUT score: *p* = 0.6835. **(G)** Hemoglobin: *p* = 0.0160. PEG, percutaneous endoscopic gastrostomy; Non-PEG, non-percutaneous endoscopic gastrostomy; BT, before treatment; W1, after one-week post-radiotherapy; W2, after 2 weeks post-radiotherapy; W3, after 3 weeks post-radiotherapy; W4, after 4 weeks post-radiotherapy; MAT, A month after treatment.

Nutritional parameters were analyzed at six time points. Independent *t*-tests revealed significant intergroup differences in weight (PEG: 54.46 kg vs. Non-PEG: 57.05 kg; *p* < 0.0001) and BMI (PEG: 21.02 kg/m^2^ vs. Non-PEG: 21.49 kg/m^2^; *p* = 0.0008). No significant differences were observed for albumin (41.92 vs. 41.31 g/L; *p* = 0.1243), lymphocyte counts (1.064 vs. 1.058 × 10^9^/L; *p* = 0.9789), cholesterol levels (4.315 vs. 4.348 mg/L; *p* = 0.8680), or CONUT scores (3.016 vs. 2.795; *p* = 0.6835). HGB levels were higher in the PEG group (120.0 vs. 114.1 g/L; *p* = 0.0160) (Figure 2).

### Overall survival

**Figure 3:**
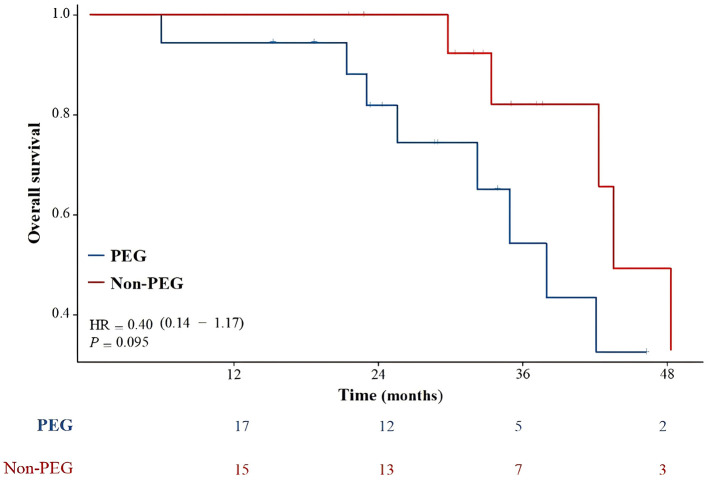
Overall survival compared PEG group with Non-PEG group. There was no statistically significant difference in survival rates between the PEG group and the Non-PEG group (*p* = 0.095).

Figure 3 illustrates the OS of the entire patient cohort. One patient was lost to follow-up after 53 months. The median follow-up was 32.7 months (range, 6–162.8). The median OS for the entire cohort was 42.3 months (range 6–162.8). The median OS was 38 months and 43.5 months for the PEG and Non-PEG groups, respectively. The 2, 3, and 4 year OS rates for the PEG group were 81.9% (95% CI: 63.2–100%), 54.3% (95% CI: 25.5–83.1%), and 32.6% (95% CI: 0.0–63.6%), respectively, while the Non-PEG group had 2, 3, and 4 year OS rates of 100% (83.0–100%), 82.1% (95% CI: 59.2–100%), and 49.2% (95% CI: 11.4–87.0%), respectively. No significant difference in OS was observed between patients who received PEG and those who did not (*p* = 0.095).

### Factors affecting OS

In univariate analysis, PEG placement prior to treatment showed no significant association with OS (HR = 0.398, 95% CI: 0.135–1.173, *p* = 0.095). A 5–10% reduction in weight loss during CCRT was significantly associated with improved survival (HR = 5.664, 95% CI: 1.075–29.846, *p* = 0.041). Changes in pre-treatment and post-treatment BMI were not statistically significantly associated with OS (Tables 3, 4). Multivariable analysis identified a pre-treatment CONUT score >4 as an independent predictor of worse OS (HR = 15.223, 95% CI: 1.935–119.783, *p* = 0.010). Pre-treatment albumin levels of 30–34.9 g/L showed a trend toward reduced OS (*p* = 0.055).

**Table 3:** Univariable analyses for variables associated with overall survival in 33 patients.

**Characteristics**	**Total (*N*)**	**Univariate analysis**	
		**HR (95% CI)**	***p*-value**
**Age (years)**			
< 65	23	Ref	
>65	10	0.807 (0.271–2.402)	0.700
**Sex**			
Male	22	Ref	
Female	11	1.302 (0.389–4.363)	0.669
**Smoke**			
never	21	Ref	
ever	12	0.611 (0.201–1.855)	0.385
**Drink**			
never	25	Ref	
ever	8	0.581 (0.156–2.169)	0.419
**T stage**			
T1	2	Ref	
T2	6	Inf	0.99
T3	10	Inf	0.998
T4	15	Inf	0.998
**N stage**			
N0	7	Ref	
N1	14	1.218 (0.296–5.024)	0.785
N2	7	1.291 (0.306–5.448)	0.728
N3	5	0.360 (0.037–3.469)	0.376
**Pathological stage**			
GX	11	Ref	
G1	1	4.224 (0.427–41.826)	0.218
G2	20	2.563 (0.780–8.423)	0.121
G3	1	0.000 (0.000–Inf)	0.998
**TNM stage**			
II	4	Ref	
III	12	3.082 (0.355–26.789)	0.308
IVA/IVB	17	2.108 (0.259–17.130)	0.485
**Induction chemotherapy**			
No	10	Ref	
Yes	23	1.372 (0.381–4.934)	0.629

HR, hazard ratio.

**Table 4:** Univariable and multivariable analyses for variables associated with overall survival in 33 patients.

**Characteristics**	**Total (*N*)**	**Univariate analysis**		**Multivariate analysis**	
		**HR (95% CI)**	***p*-value**	**HR (95% CI)**	***p*-value**
**PEG**					
Yes	18	Ref		Ref	
No	15	0.398 (0.135–1.173)	0.095	0.398 (0.135–1.173)	0.095
**Weight loss (%)**					
< 5	27	Ref			
5–10	4	5.664 (1.075–29.846)	**0.041**		
>10	2	2.426 (0.282–20.837)	0.419		
**Before treatment BMI (kg/m** ^2^ **)**					
< 18.5	7	Ref			
18.5–24.99	21	0.767 (0.260–2.257)	0.629		
25–29.99	4	Inf	0.999		
≥30	1	Inf	0.999		
**After treatment BMI (kg/m** ^2^ **)**					
< 18.5	6	Ref			
18.5–24.99	24	0.413 (0.123–1.384)	0.152		
25–29.99	3	Inf	0.999		
**Albumin infusion during treatment**					
No	26	Ref			
Yes	7	2.190 (0.430–11.166)	0.346		
**Decreased Hemoglobin class during CCRT (%)**					
< 5	19	Ref			
5–10	2	2.106 (0.437–10.152)	0.353		
>10	12	0.723 (0.221–2.364)	0.591		
**Pre-treatment albumin (g/L)**					
≥35	30	Ref			
30–34.9	2	9.259 (0.955–89.765)	0.055		
< 25	1	1.408 (0.176–11.284)	0.747		
**Albumin after treatment (g/L)**					
≥35	31	Ref			
30–34.9	2	3.530 (0.392–31.797)	0.261		
**Pre-treatment lymphocyte (10** ^9^ **/L)**					
>1.6	17	Ref			
1.2–1.59	11	1.467 (0.450–4.786)	0.525		
0.8–1.19	4	1.034 (0.216–4.946)	0.967		
< 0.8	1	0.000 (0.000–Inf)	0.998		
**Lymphocyte after treatment (10** ^9^ **/L)**					
>1.6	6	Ref			
1.2–1.59	9	0.425 (0.070–2.579)	0.352		
0.8–1.19	9	0.894 (0.151–5.289)	0.901		
< 0.8	9	0.652 (0.110–3.879)	0.639		
**Pretreatment cholesterol (mmol/L)**					
>4.68	13	Ref			
3.64-4.67	14	0.949 (0.296–3.044)	0.929		
2.6-3.63	4	3.109 (0.554–17.460)	0.198		
< 2.6	2	8.995 (0.817–99.082)	0.073		
**Post-treatment cholesterol (mmol/L)**					
>4.68	15	Ref			
3.64–4.67	12	1.080 (0.314–3.719)	0.903		
2.6–3.63	4	1.117 (0.226–5.531)	0.892		
< 2.6	2	7.830 (0.747–82.113)	0.086		
**Pre-treatment CONUT score**					
0–1 (good)	18	Ref			
2–3 (fair)	12	0.999 (0.321–3.104)	0.998	0.999 (0.321–3.104)	0.998
≥4 (poor)	3	15.223 (1.935–119.783)	**0.010**	15.223 (1.935–119.783)	**0.010**
**After treatment CONUT score**					
0–1 (good)	7	Ref			
2–3 (fair)	18	0.767 (0.197–2.986)	0.702		
≥4 (poor)	8	1.608 (0.346–7.475)	0.545		

CCRT, concurrent chemoradiotherapy; BMI, body mass index; CONUT, controlling nutritional status; Statistically significant values (p < 0.05) are given in bold.

## Discussion

The aim of this study was to examine whether the pre-treatment implantation of PEG influences the nutritional status and OS of patients. Patient variables such as nutritional status (weight, BMI, HGB, CONUT score) were analyzed for their statistical association with PEG implantation. Contrary to expectations, the study findings revealed no significant difference in OS between the PEG and Non-PEG groups, suggesting that the decision to implant PEG before treatment did not impact patient outcomes.

Despite prior evidence supporting PEG's role in maintaining nutritional status (27–31), our study revealed paradoxical outcomes. While significant differences in baseline weight (*p* = 0.0001) and BMI (*p* = 0.0008) were observed between PEG and Non-PEG groups, Non-PEG patients exhibited higher baseline values—a finding attributable to selection bias. PEG insertion was typically indicated for patients with swallowing difficulties or significant comorbidities, rather than routinely performed. Therefore, patients undergoing PEG insertion may have already experienced significant weight loss prior to treatment, which could introduce bias into the study's findings. Furthermore, our findings revealed that the proportion of patients with a BMI < 18.5 kg/m^2^ decreased from 5/18 (27.3%) to 3/18 (16.7%) in the PEG group, suggesting a positive impact of PEG on nutritional status. In contrast, the Non-PEG group exhibited an increase in the proportion of patients with a BMI < 18.5 kg/m^2^, from 2/16 (12.5%) to 4/16 (25.0%). This suggests that PEG may play a beneficial role in improving BMI and potentially mitigating malnutrition in these patients. However, the existing literature remains unclear regarding the impact of weight loss and BMI on outcomes in patients with EC.

Our analysis revealed a statistically significant intergroup difference in HGB levels (*p* = 0.0160), but no definitive association with prognosis was observed. This may be attributed to two confounding factors. First, 50% of our cohort consisted of stage IV patients, where tumor-driven systemic effects, such as chronic hemorrhage and chemotherapy-induced myelosuppression, likely overshadow the influence of baseline hematologic parameters (32). Second, the absence of HGB stratification analysis in our study limited the ability to detect potential threshold-dependent effects. Previous studies have suggested that low HGB levels are associated with poorer outcomes in various cancer types (33, 34). However, other research has reported no significant association between HGB levels and prognosis in esophageal cancer treatment (35, 36). These conflicting findings highlight the complexity of the relationship between HGB and prognosis, which is influenced by disease stage, treatment effects, and the lack of stratified analysis.

Although baseline nutritional status may vary between groups, the overall CONUT score did not significantly differ based on PEG implantation. One limitation of the CONUT score is its reliance on serum biomarkers, which do not capture the complex effects of PEG tube feeding on nutrition. While PEG tube feeding may temporarily improve intake, overall nutritional status does not show significant improvement due to factors such as inflammation, metabolic disturbances, and gastrointestinal dysfunction. Therefore, a more comprehensive nutritional assessment, incorporating additional biomarkers, may be needed to better evaluate the impact of PEG feeding in CEC patients.

In our multivariate analysis, we found that a weight loss rate of 5–10% and higher CONUT scores (≥4) were significantly associated with poorer survival outcomes, emphasizing the critical role of nutritional status in predicting OS. Based on this, we further explored the role of PEG tube placement in improving nutritional outcomes and survival.

Interestingly, no statistically significant difference in OS was found between the PEG and Non-PEG groups. We propose three potential explanations for this. First, chemoradiotherapy has been shown to alleviate dysphagia and improve eating difficulties in patients with CEC, potentially reducing the need for PEG insertion (37). This could explain the lack of significant weight changes in the Non-PEG group during treatment. Second, a higher proportion of patients in the PEG group were classified as T-stage 4, which is associated with poorer prognosis, potentially offsetting any survival benefit from PEG placement. Third, patients in the Non-PEG group had better baseline nutritional status, which may have contributed to a relatively better prognosis despite the absence of PEG placement.

Overall, our study suggests that prophylactic PEG placement is most beneficial for patients with moderate to severe weight loss prior to chemoradiotherapy. For patients without additional risk factors, PEG placement may not provide significant survival benefits. These findings highlight the importance of assessing baseline nutritional status when considering interventions aimed at improving survival outcomes in patients with ESCC (38).

The present study has several limitations. Firstly, the analysis was based on a relatively small patient cohort, which may have impacted the accuracy of the results. CEC is a rare disease with a low incidence rate, and the stringent inclusion criteria, which excluded patients with distant metastasis or comorbid cancers, further limited the cohort size. These restrictions led to baseline differences between the two patient groups, potentially affecting the comparison and interpretation of the results. Nevertheless, considering the limited research available on this patient population, this study provides valuable insights into enhancing the understanding of this rare group. Secondly, the retrospective nature of the study, conducted at a single institution introduces potential biases. Future multicenter studies with larger sample sizes could help correct sample bias. Thirdly, the lack of comprehensive data on reasons for PEG placement before treatment initiation hindered a detailed analysis of the observed relationship between pretreatment PEG placement and patient outcomes. Additionally, the study did not document adverse events related to enteral feeding during the treatment period, such as PEG dislodgement or feed leakage at the gastrostomy site, which could have influenced final weight differences. Importantly, all patients, regardless of whether they experienced adverse events, were included in the analysis, which may impact the overall outcome.

Given the potential impact of nutritional status on patient outcomes, it is reasonable to consider interventions that could improve nutritional support. Consequently, while the direct causal link between PEG and survival remains speculative, the prognostic significance of its presence—particularly in patients with poor nutritional status—provides valuable information for clinicians managing these patients.

## Conclusions

Our results indicate that pre-treatment PEG placement had no significant impact on patients' nutritional parameters. Stratified analysis showed that patients with weight loss < 5% during chemoradiotherapy had better survival rates, while CONUT scores ≥4 were identified as an independent negative prognostic factor for OS.

## Data Availability

The raw data supporting the conclusions of this article will be made available by the authors, without undue reservation.

## References

[1] Sung H Ferlay J Siegel RL Laversanne M Soerjomataram I Jemal A. Global Cancer Statistics 2020: GLOBOCAN estimates of incidence and mortality worldwide for 36 cancers in 185 countries. CA Cancer J Clin. (2021) 71:209–49. 10.3322/caac.21660 33538338

[2] Tong DK Law S Kwong DL Wei WI Ng RW Wong KH. Current management of cervical esophageal cancer. World J Surg. (2011) 35:600–7. 10.1007/s00268-010-0876-7 21161656

[3] Lagergren J Smyth E Cunningham D Lagergren P. Oesophageal cancer. Lancet. (2017) 390:2383–96. 10.1016/S0140-6736(17)31462-9 28648400

[4] Van De Voorde L Larue RT Pijls M Buijsen J Troost EG Berbée M. A qualitative synthesis of the evidence behind elective lymph node irradiation in oesophageal cancer. Radiother Oncol. (2014) 113:166–74. 10.1016/j.radonc.2014.11.010 25465727

[5] Riccardi D Allen K. Nutritional management of patients with esophageal and esophagogastric junction cancer. Cancer Control. (1999) 6:64–72. 10.1177/107327489900600106 10758536

[6] Jordan T Mastnak DM Palamar N Kozjek NR. Nutritional therapy for patients with esophageal cancer. Nutr Cancer. (2018) 70:23–9. 10.1080/01635581.2017.1374417 29016197

[7] Hébuterne X Lemarié E Michallet M de Montreuil CB Schneider SM Goldwasser F. Prevalence of malnutrition and current use of nutrition support in patients with cancer. JPEN J Parenter Enteral Nutr. (2014) 38:196–204. 10.1177/0148607113502674 24748626

[8] Lloyd S Chang BW. Current strategies in chemoradiation for esophageal cancer. J Gastrointest Oncol. (2014) 5:156–65. 24982764 10.3978/j.issn.2078-6891.2014.033 PMC4074950

[9] Meier RF Forbes A. Basics in clinical medical nutrition. Nestle Nutr Inst Workshop Ser. (2015) 82:1–16. 10.1159/000381997 26544878

[10] Van Cutsem E Arends J. The causes and consequences of cancer-associated malnutrition. Eur J Oncol Nurs. (2005) 9:S51–63. 10.1016/j.ejon.2005.09.007 16437758

[11] Han H Pan M Tao Y Liu R Huang Z Piccolo K. Early enteral nutrition is associated with faster post-esophagectomy recovery in Chinese esophageal cancer patients: a retrospective cohort study. Nutr Cancer. (2018) 70:221–8. 10.1080/01635581.2018.1412477 29313724

[12] Gauderer MW Ponsky JL Izant RJ. Gastrostomy without laparotomy: a percutaneous endoscopic technique. J Pediatr Surg. (1980) 15:872–5. 10.1016/S0022-3468(80)80296-X 6780678

[13] Gauderer MW. Percutaneous endoscopic gastrostomy-20 years later: a historical perspective. J Pediatr Surg. (2001) 36:217–9. 10.1053/jpsu.2001.20058 11150469

[14] Burney RE Bryner BS. Safety and long-term outcomes of percutaneous endoscopic gastrostomy in patients with head and neck cancer. Surg Endosc. (2015) 29:3685–9. 10.1007/s00464-015-4126-9 25740644

[15] Wermker K Jung S Huppmeier L Joos U Kleinheinz J. Prediction model for early percutaneous endoscopic gastrostomy (PEG) in head and neck cancer treatment. Oral Oncol. (2012) 48:355–60. 10.1016/j.oraloncology.2011.11.005 22154128

[16] Tabrizi R Hosseinpour S Taghizadeh F. Feeding in oral cancer patients after massive ablative surgery: percutaneous endoscopic gastrostomy or nasogastric tube. J Craniofac Surg. (2016) 27:1010–1. 10.1097/SCS.0000000000002662 27228377

[17] Margolis M Alexander P Trachiotis GD Gharagozloo F Lipman T. Percutaneous endoscopic gastrostomy before multimodality therapy in patients with esophageal cancer. Ann Thorac Surg. (2003) 76:1694–7; discussion 1697–8. 10.1016/S0003-4975(02)04890-7 14602314

[18] Grilo A Santos CA Fonseca J. Percutaneous endoscopic gastrostomy for nutritional palliation of upper esophageal cancer unsuitable for esophageal stenting. Arq Gastroenterol. (2012) 49:227–31. 10.1590/S0004-28032012000300012 23011248

[19] Grant DG Bradley PT Pothier DD Bailey D Caldera S Baldwin DL. Complications following gastrostomy tube insertion in patients with head and neck cancer: a prospective multi-institution study, systematic review and meta-analysis. Clin Otolaryngol. (2009) 34:103–12. 10.1111/j.1749-4486.2009.01889.x 19413607

[20] Zuercher BF Grosjean P Monnier P. Percutaneous endoscopic gastrostomy in head and neck cancer patients: indications, techniques, complications and results. Eur Arch Otorhinolaryngol. (2011) 268:623–9. 10.1007/s00405-010-1412-y 21046412

[21] Pohar S Demarcantonio M Whiting P Crandley E Wadsworth J Karakla D. Percutaneous endoscopic gastrostomy tube dependence following chemoradiation in head and neck cancer patients. Laryngoscope. (2015) 125:1366–71. 10.1002/lary.25117 25647161

[22] Odelli C Burgess D Bateman L Hughes A Ackland S Gillies J. Nutrition support improves patient outcomes, treatment tolerance and admission characteristics in oesophageal cancer. Clin Oncol (R Coll Radiol). (2005) 17:639–45. 10.1016/j.clon.2005.03.015 16372491

[23] Matsumoto A Yuda M Tanaka Y Tanishima Y Yano F Nishikawa K. Efficacy of percutaneous endoscopic gastrostomy for patients with esophageal cancer during preoperative therapy. Anticancer Res. (2019) 39:4243–8. 10.21873/anticanres.13586 31366512

[24] Langius JA Bakker S Rietveld DH Kruizenga HM Langendijk JA Weijs PJ. Critical weight loss is a major prognostic indicator for disease-specific survival in patients with head and neck cancer receiving radiotherapy. Br J Cancer. (2013) 109:1093–9. 10.1038/bjc.2013.458 23928661 PMC3778304

[25] Ignacio de Ulibarri J., González-Madroño A, de Villar NG, González P, González B, Mancha A, et al. CONUT: a tool for controlling nutritional status. First validation in a hospital population. Nutr Hosp. (2005) 20, 38–45. 15762418

[26] Suzuki H Ito M Takemura K Kobayashi S Kataoka M Iida N. The Controlling Nutritional Status (CONUT) score is a prognostic biomarker in advanced urothelial carcinoma patients treated with first-line platinum-based chemotherapy. Bladder Cancer. (2021) 7:13–21. 10.3233/BLC-200354 38993214 PMC11181873

[27] Locher JL Bonner JA Carroll WR Caudell JJ Keith JN Kilgore ML. Prophylactic percutaneous endoscopic gastrostomy tube placement in treatment of head and neck cancer: a comprehensive review and call for evidence-based medicine. JPEN J Parenter Enteral Nutr. (2011) 35:365–74. 10.1177/0148607110377097 21527598

[28] Mercuri A Lim Joon D Wada M Rolfo A Khoo V. The effect of an intensive nutritional program on daily set-up variations and radiotherapy planning margins of head and neck cancer patients. J Med Imaging Radiat Oncol. (2009) 53:500–5. 10.1111/j.1754-9485.2009.02105.x 19788487

[29] Chang JH Gosling T Larsen J Powell S Scanlon R Chander S. Prophylactic gastrostomy tubes for patients receiving radical radiotherapy for head and neck cancers: a retrospective review. J Med Imaging Radiat Oncol. (2009) 53:494–9. 10.1111/j.1754-9485.2009.02103.x 19788486

[30] Chen AM Li BQ Lau DH Farwell DG Luu Q Stuart K. Evaluating the role of prophylactic gastrostomy tube placement prior to definitive chemoradiotherapy for head and neck cancer. Int J Radiat Oncol Biol Phys. (2010) 78:1026–32. 10.1016/j.ijrobp.2009.09.036 20231073

[31] Dong J Dai Z Cao F Zhang W Zhang T Chen X. Effects of PEG in patients with esophageal squamous cell carcinoma during concurrent chemoradiotherapy: a prospective study. Gastrointest Endosc. (2023) 98:901–10 e903. 10.1016/j.gie.2023.04.2094 37150411

[32] Schwartz RN. Anemia in patients with cancer: incidence, causes, impact, management, and use of treatment guidelines and protocols. Am J Health Syst Pharm. (2007) 64, S5–13; quiz S28–30. 10.2146/ajhp060601 17244886

[33] Wang B Jiang XW Tian DL Zhou N Geng W. Combination of Haemoglobin and Prognostic nutritional index predicts the prognosis of postoperative radiotherapy for esophageal squamous cell carcinoma. Cancer Manag Res. (2020) 12:8589–97. 10.2147/CMAR.S266821 32982451 PMC7509334

[34] Coradduzza D Medici S Chessa C Zinellu A Madonia M Angius A. Assessing the predictive power of the hemoglobin/red cell distribution width ratio in cancer: a systematic review and future directions. Medicina (Kaunas). (2023) 59:2124. 10.3390/medicina59122124 38138227 PMC10744746

[35] Zhang F Chen Z Wang P Hu X Gao Y He J. Combination of platelet count and mean platelet volume (COP-MPV) predicts postoperative prognosis in both resectable early and advanced stage esophageal squamous cell cancer patients. Tumour Biol. (2016) 37:9323–31. 10.1007/s13277-015-4774-3 26779631 PMC4990601

[36] Jokela EMK Kauppila JH on on behalf of the FINEGO group. Preoperative hemoglobin count and prognosis of esophageal cancer, a population-based nationwide study in Finland. Eur J Surg Oncol. (2022) 48:548–52. 10.1016/j.ejso.2021.08.020 34420826

[37] Cao C Luo J Gao L Xu G Yi J Huang X. Definitive radiotherapy for cervical esophageal cancer. Head Neck. (2015) 37:151–5. 10.1002/hed.23572 24347470

[38] Brown T Banks M Hughes B Kenny L Lin C Bauer J. Protocol for a randomized controlled trial of early prophylactic feeding via gastrostomy versus standard care in high risk patients with head and neck cancer. BMC Nurs. (2014) 13:17. 10.1186/1472-6955-13-17 25002833 PMC4083037

