# Supplementary material for: The impact of percutaneous endoscopic gastrostomy on nutritional status and survival in cervical esophageal cancer patients undergoing chemoradiotherapy
Source: Front Nutr. 2025 Jun 25;12:1521239. doi: 10.3389/fnut.2025.1521239 (PMC12237668; doi:10.3389/fnut.2025.1521239)
Supplement: Supplementary file 1 [file Table_1.docx]

Supplementary Material

# Supplementary Tables

**Supplementary table 1. Effect of PEG on body weight in 6 time periods.**

|  |  | **BT** | **W1** | **W2** | **W3** | **W4** | **MAT** |
| --- | --- | --- | --- | --- | --- | --- | --- |
| **PEG group** | **1** | **52.0** | **48.5** | **46.0** | **48.0** | **48.0** | **53.0** |
|  | **2** | **32.5** | **32.5** | **32.0** | **30.0** | **30.0** | **33.0** |
|  | **3** | 60.4 | 58.9 | 57.6 | 57.0 | 56.6 | 56.3 |
|  | **4** | **51.9** | 53.4 | 54.8 | 53.1 | 54.0 | 55.0 |
|  | **5** | **55.3** | **53.4** | **51.4** | **51.0** | **50.0** | **52.0** |
|  | **6** | **70.0** | **67.0** | **68.0** | **67.3** | **66.0** | **68.3** |
|  | **7** | 52.9 | 48.2 | 48.0 | 50.0 | 49.0 | 51.0 |
|  | **8** | **57.9** | **58.6** | **57.4** | **58.8** | **58.8** | **63.0** |
|  | **9** | **51.3** | **54.8** | **54.5** | **54.6** | **54.0** | **56.0** |
|  | **10** | **56.9** | **56.4** | **55.6** | **54.4** | **54.0** | **53.5** |
|  | **11** | **64.5** | **63.7** | **62.6** | **62.0** | **61.9** | **62.5** |
|  | **12** | **51.3** | **51.0** | **51.3** | **50.6** | **51.0** | **51.9** |
|  | **13** | **52.0** | **52.3** | **53.2** | **52.4** | **53.2** | **53.6** |
|  | **14** | **64.3** | **62.8** | **63.4** | **63.9** | **64.0** | **63.6** |
|  | **15** | **50.5** | **50.5** | **51.0** | **51.0** | **52.0** | **53.0** |
|  | **16** | **60.0** | **57.1** | **61.0** | **56.0** | **58.2** | **59.8** |
|  | **17** | **59.7** | **60.0** | **57.5** | **62.0** | **63.0** | **60.0** |
|  | **18** | **45.5** | **49.0** | **50.0** | **50.0** | **49.0** | **49.O** |
| **Non-PEG group** | **1** | **62.1** | **62.1** | **61.6** | **61.5** | **61.4** | **61.0** |
|  | **2** | **78.0** | **76.0** | **74.5** | **73.0** | **71.3** | **69.0** |
|  | **3** | **49.5** | **51.8** | **51.5** | **50.3** | **50.5** | **52.0** |
|  | **4** | **52.4** | **54.0** | **51.6** | **50.2** | **50.0** | **52.0** |
|  | **5** | **62.0** | **61.9** | **62.5** | **61.0** | **61.1** | **62.6** |
|  | **6** | **69.8** | **70.6** | **70.0** | **69.7** | **70.0** | **70.1** |
|  | **7** | **58.5** | **59.3** | **58.6** | **58.1** | **58.5** | **60.0** |
|  | **8** | **62.8** | **61.1** | **60.5** | **60.6** | **61.0** | **63.0** |
|  | **9** | **60.9** | **63.9** | **64.0** | **64.2** | **63.5** | **64.8** |
|  | **10** | **38.5** | **39.1** | **39.0** | **38.5** | **38.9** | **38.7** |
|  | **11** | **62.9** | **58.0** | **58.2** | **58.0** | **58.1** | **58.4** |
|  | **12** | **53.5** | **53.9** | **54.0** | **54.4** | **52.4** | **48.0** |
|  | **13** | **61.8** | **61.0** | **60.0** | **61.2** | **59.0** | **60.8** |
|  | **14** | **45.0** | **46.0** | **42.5** | **45.0** | **44.0** | **43.4** |
|  | **15** | **60.0** | **62.8** | **63.5** | **63.3** | **63.5** | **64.5** |
|  | **16** | **39.8** | **41.1** | **41.0** | **41.2** | **40.6** | **41.3** |

**Supplementary table 2. Effect of PEG on BMI in 6 time periods.**

|  |  | **BT** | **W1** | **W2** | **W3** | **W4** | **MAT** |
| --- | --- | --- | --- | --- | --- | --- | --- |
| **PEG group** | **1** | **17.99** | **16.78** | **15.92** | **16.61** | **16.61** | **18.34** |
|  | **2** | **15.14** | **15.14** | **14.91** | **13.98** | **13.98** | **15.38** |
|  | **3** | **23.89** | **23.30** | **22.78** | **22.55** | **22.39** | **22.27** |
|  | **4** | **23.38** | **24.05** | **24.68** | **23.92** | **24.32** | **24.55** |
|  | **5** | **21.33** | **20.60** | **19.83** | **19.68** | **19.29** | **20.06** |
|  | **6** | **25.10** | **24.00** | **24.40** | **24.49** | **24.74** | **24.49** |
|  | **7** | **19.67** | **17.92** | **17.85** | **18.59** | **18.22** | **18.96** |
|  | **8** | **23.49** | **23.77** | **23.29** | **23.85** | **23.85** | **25.56** |
|  | **9** | **15.83** | **16.91** | **16.82** | **16.85** | **16.67** | **17.28** |
|  | **10** | **21.03** | **20.84** | **20.55** | **20.10** | **19.96** | **19.77** |
|  | **11** | **25.51** | **25.20** | **24.76** | **24.52** | **24.48** | **24.70** |
|  | **12** | **23.74** | **23.60** | **23.74** | **23.42** | **23.60** | **24.02** |
|  | **13** | **20.31** | **20.43** | **20.78** | **20.47** | **20.78** | **20.94** |
|  | **14** | **26.94** | **26.31** | **26.56** | **26.77** | **26.81** | **26.64** |
|  | **15** | **17.89** | **17.89** | **18.07** | **18.07** | **18.42** | **18.78** |
|  | **16** | **21.26** | **20.23** | **21.61** | **19.84** | **20.62** | **21.19** |
|  | **17** | **21.15** | **21.26** | **20.37** | **21.97** | **22.32** | **21.26** |
|  | **18** | **17.66** | **19.14** | **19.53** | **19.53** | **19.14** | **19.14** |
| **Non-PEG group** | **1** | **23.96** | **23.96** | **23.76** | **23.73** | **23.69** | **23.53** |
|  | **2** | **32.03** | **31.23** | **30.61** | **30.00** | **29.30** | **28.35** |
|  | **3** | **18.59** | **19.50** | **19.38** | **18.93** | **19.01** | **19.57** |
|  | **4** | **22.98** | **23.68** | **22.63** | **22.02** | **21.93** | **22.81** |
|  | **5** | **24.22** | **24.18** | **24.41** | **23.83** | **23.87** | **24.45** |
|  | **6** | **23.59** | **23.86** | **23.66** | **23.56** | **23.66** | **23.70** |
|  | **7** | **25.49** | **25.84** | **25.53** | **25.31** | **25.49** | **26.14** |
|  | **8** | **20.39** | **19.84** | **19.64** | **19.68** | **19.81** | **20.45** |
|  | **9** | **22.51** | **23.61** | **23.65** | **23.72** | **23.47** | **23.95** |
|  | **10** | **17.11** | **17.38** | **17.33** | **17.11** | **17.29** | **17.20** |
|  | **11** | **21.14** | **19.49** | **19.56** | **19.49** | **19.53** | **19.63** |
|  | **12** | **18.62** | **18.76** | **18.8** | **18.93** | **18.24** | **16.71** |
|  | **13** | **20.41** | **20.15** | **19.82** | **20.21** | **19.49** | **20.08** |
|  | **14** | **18.73** | **19.15** | **17.69** | **18.73** | **18.31** | **18.06** |
|  | **15** | **21.91** | **22.93** | **23.18** | **23.11** | **23.18** | **23.55** |
|  | **16** | **13.85** | **14.31** | **14.27** | **14.34** | **14.13** | **14.38** |

**Supplementary table 3. Effect of PEG on albumin in 6 time periods.**

|  |  | **BT** | **W1** | **W2** | **W3** | **W4** | **MAT** |
| --- | --- | --- | --- | --- | --- | --- | --- |
| **PEG group** | **1** | **43.9** | **36.4** | **37.6** | **40.1** | **41.1** | **44.1** |
|  | **2** | **44.1** | **46.4** | **43.1** | **39.7** | **41.2** | **44.1** |
|  | **3** | **43.9** | **46.6** | **48.3** | **42.6** | **42.2** | **41.2** |
|  | **4** | **38.7** | **41.4** | **40.6** | **42.3** | **38.9** | **42.9** |
|  | **5** | **36.6** | **40.0** | **39.7** | **39.1** | **45.2** | **45.2** |
|  | **6** | **38.6** | **37.9** | **39.1** | **38.8** | **38.0** | **39.2** |
|  | **7** | **17.7** | **32.7** | **34.8** | **34.2** | **34.1** | **39.1** |
|  | **8** | **39.7** | **42.2** | **45.1** | **44.0** | **40.0** | **44.3** |
|  | **9** | **39.6** | **43.0** | **43.0** | **41.7** | **39.1** | **45.9** |
|  | **10** | **45.3** | **42.4** | **49.5** | **43.3** | **42.5** | **45.9** |
|  | **11** | **41.0** | **44.6** | **43.5** | **42.6** | **43.0** | **45.0** |
|  | **12** | **39.8** | **39.6** | **40.4** | **42.9** | **44.6** | **46.0** |
|  | **13** | **39.1** | **47.7** | **44.5** | **41.0** | **44.2** | **40.5** |
|  | **14** | **44.8** | **41.9** | **42.8** | **39.8** | **40.0** | **40.8** |
|  | **15** | **43.4** | **50.5** | **44.4** | **46.6** | **42.6** | **41.9** |
|  | **16** | **45.2** | **40.6** | **44.8** | **45.6** | **36.6** | **45.8** |
|  | **17** | **44.7** | **43.9** | **44.3** | **43.8** | **38.5** | **42.7** |
|  | **18** | **46.2** | **44.9** | **40.9** | **44.6** | **44.0** | **39.8** |
| **Non-PEG group** | **1** | **42.2** | **39.8** | **46.3** | **43.2** | **44.3** | **46.0** |
|  | **2** | **46.6** | **45.6** | **45.3** | **42.8** | **41.9** | **40.0** |
|  | **3** | **36.7** | **42.3** | **37.4** | **41.0** | **36.6** | **41.2** |
|  | **4** | **34.4** | **41.1** | **41.7** | **34.0** | **35.1** | **40.7** |
|  | **5** | **45.5** | **42.3** | **39.6** | **50.5** | **44.5** | **44.3** |
|  | **6** | **44.5** | **45.1** | **48.8** | **47.7** | **46.0** | **46.1** |
|  | **7** | **37.5** | **44.8** | **42.4** | **38.9** | **39.1** | **43.3** |
|  | **8** | **41.3** | **37.5** | **38.3** | **35.0** | **34.1** | **39.8** |
|  | **9** | **39.1** | **40.8** | **40.7** | **40.4** | **40.7** | **42.4** |
|  | **10** | **49.6** | **40.4** | **38.2** | **42.6** | **43.0** | **40.1** |
|  | **11** | **48.6** | **45.7** | **41.5** | **39.2** | **39.1** | **44.3** |
|  | **12** | **44.2** | **40.9** | **41.2** | **41.0** | **39.0** | **36.7** |
|  | **13** | **43.0** | **40.7** | **38.0** | **38.1** | **38.7** | **43.0** |
|  | **14** | **39.4** | **44.1** | **39.8** | **42.2** | **40.0** | **41.5** |
|  | **15** | **37.3** | **40.4** | **40.9** | **41.5** | **43.0** | **45.0** |
|  | **16** | **31.4** | **36.1** | **36.9** | **42.9** | **42.9** | **36.5** |

**Supplementary table 4. Effect of PEG on lymphocyte count in 6 time periods.**

|  |  | **BT** | **W1** | **W2** | **W3** | **W4** | **MAT** |
| --- | --- | --- | --- | --- | --- | --- | --- |
| **PEG group** | **1** | **3.01** | **1.04** | **0.58** | **0.72** | **0.81** | **1.95** |
|  | **2** | **1.25** | **1.46** | **0.29** | **0.17** | **0.67** | **0.84** |
|  | **3** | **1.04** | **1.19** | **2.05** | **0.85** | **0.66** | **1.29** |
|  | **4** | **1.49** | **1.49** | **1.15** | **0.30** | **0.31** | **0.84** |
|  | **5** | **1.69** | **0.94** | **1.20** | **0.47** | **0.47** | **1.54** |
|  | **6** | **1.48** | **0.63** | **0.40** | **0.51** | **0.82** | **0.87** |
|  | **7** | **2.11** | **0.92** | **0.74** | **0.86** | **0.86** | **1.66** |
|  | **8** | **1.00** | **1.25** | **0.70** | **0.3** | **0.56** | **1.24** |
|  | **9** | **1.53** | **1.76** | **1.91** | **1.09** | **0.60** | **1.23** |
|  | **10** | **2.00** | **1.04** | **1.56** | **0.37** | **0.35** | **0.90** |
|  | **11** | **1.97** | **2.30** | **1.16** | **0.62** | **0.90** | **1.66** |
|  | **12** | **1.29** | **1.31** | **0.77** | **0.47** | **0.51** | **1.20** |
|  | **13** | **2.15** | **1.26** | **0.76** | **0.41** | **0.38** | **0.84** |
|  | **14** | **2.08** | **0.71** | **0.66** | **0.52** | **0.56** | **1.26** |
|  | **15** | **1.95** | **0.77** | **0.45** | **0.50** | **0.24** | **0.58** |
|  | **16** | **1.55** | **1.57** | **1.74** | **1.18** | **0.57** | **1.28** |
|  | **17** | **1.72** | **0.79** | **0.69** | **0.22** | **0.65** | **1.68** |
|  | **18** | **2.08** | **1.04** | **0.61** | **0.32** | **0.60** | **1.14** |
| **Non-PEG group** | **1** | **2.17** | **0.99** | **0.95** | **0.57** | **0.54** | **1.22** |
|  | **2** | **1.86** | **2.45** | **0.69** | **0.46** | **0.20** | **1.61** |
|  | **3** | **1.09** | **1.44** | **0.78** | **0.53** | **0.31** | **1.70** |
|  | **4** | **1.60** | **1.68** | **0.95** | **0.20** | **0.57** | **1.60** |
|  | **5** | **0.95** | **0.94** | **0.46** | **0.44** | **0.34** | **0.82** |
|  | **6** | **1.35** | **1.01** | **1.18** | **0.94** | **0.88** | **1.19** |
|  | **7** | **2.31** | **0.55** | **0.75** | **0.45** | **0.69** | **1.63** |
|  | **8** | **1.95** | **0.46** | **0.45** | **0.55** | **0.88** | **2.99** |
|  | **9** | **1.38** | **0.97** | **0.63** | **0.31** | **0.34** | **1.10** |
|  | **10** | **1.55** | **1.81** | **1.01** | **0.78** | **0.53** | **0.81** |
|  | **11** | **3.16** | **1.78** | **0.85** | **0.56** | **0.26** | **1.27** |
|  | **12** | **1.28** | **1.00** | **0.32** | **0.22** | **0.85** | **0.72** |
|  | **13** | **1.79** | **1.14** | **0.80** | **0.51** | **1.11** | **1.80** |
|  | **14** | **0.80** | **0.69** | **0.54** | **0.48** | **0.33** | **2.48** |
|  | **15** | **1.85** | **1.81** | **1.12** | **0.99** | **0.46** | **1.25** |
|  | **16** | **0.36** | **1.50** | **1.19** | **0.80** | **0.92** | **0.57** |

**Supplementary table 5. Effect of PEG on cholesterol in 6 time periods**

|  |  | **BT** | **W1** | **W2** | **W3** | **W4** | **MAT** |
| --- | --- | --- | --- | --- | --- | --- | --- |
| **PEG group** | **1** | **3.95** | **2.77** | **2.85** | **3.89** | **3.88** | **5.13** |
|  | **2** | **4.80** | **4.23** | **3.85** | **3.12** | **4.55** | **5.35** |
|  | **3** | **5.26** | **5.00** | **5.08** | **4.36** | **4.10** | **6.23** |
|  | **4** | **6.17** | **6.59** | **6.74** | **6.07** | **4.54** | **8.02** |
|  | **5** | **2.46** | **2.63** | **2.48** | **2.57** | **2.03** | **4.06** |
|  | **6** | **4.25** | **2.85** | **3.24** | **3.15** | **3.51** | **3.58** |
|  | **7** | **1.58** | **2.14** | **2.18** | **2.44** | **2.34** | **2.90** |
|  | **8** | **3.05** | **3.56** | **2.85** | **3.88** | **3.96** | **4.44** |
|  | **9** | **3.92** | **3.87** | **4.31** | **4.39** | **3.91** | **4.88** |
|  | **10** | **4.61** | **3.88** | **3.86** | **2.89** | **2.88** | **4.44** |
|  | **11** | **4.86** | **4.69** | **3.09** | **4.51** | **4.44** | **4.55** |
|  | **12** | **4.49** | **4.30** | **3.31** | **3.25** | **3.95** | **5.60** |
|  | **13** | **5.66** | **5.83** | **5.77** | **5.07** | **5.35** | **4.23** |
|  | **14** | **4.56** | **3.57** | **3.63** | **3.95** | **4.11** | **3.70** |
|  | **15** | **4.66** | **5.31** | **5.71** | **5.83** | **5.66** | **4.18** |
|  | **16** | **3.53** | **4.38** | **4.61** | **4.45** | **3.34** | **4.58** |
|  | **17** | **4.98** | **5.04** | **4.52** | **4.74** | **4.70** | **6.91** |
|  | **18** | **6.00** | **5.35** | **4.76** | **4.47** | **2.85** | **4.65** |
| **Non-PEG group** | **1** | **4.98** | **4.60** | **4.96** | **4.83** | **4.60** | **4.40** |
|  | **2** | **5.52** | **5.50** | **5.08** | **4.99** | **4.92** | **4.45** |
|  | **3** | **4.87** | **5.48** | **5.25** | **3.26** | **3.77** | **5.60** |
|  | **4** | **3.89** | **5.28** | **4.02** | **3.26** | **3.80** | **5.48** |
|  | **5** | **3.73** | **3.43** | **3.53** | **4.03** | **4.02** | **4.27** |
|  | **6** | **5.50** | **4.83** | **5.64** | **4.93** | **4.68** | **6.34** |
|  | **7** | **3.29** | **3.79** | **2.93** | **2.35** | **3.00** | **5.96** |
|  | **8** | **4.23** | **4.60** | **5.02** | **4.89** | **5.00** | **5.87** |
|  | **9** | **4.70** | **4.76** | **4.34** | **4.59** | **4.92** | **4.71** |
|  | **10** | **4.07** | **4.02** | **3.26** | **2.34** | **2.73** | **4.11** |
|  | **11** | **5.02** | **4.52** | **4.33** | **4.33** | **5.78** | **4.16** |
|  | **12** | **4.48** | **4.52** | **3.36** | **3.40** | **3.51** | **3.05** |
|  | **13** | **3.98** | **3.67** | **2.95** | **3.00** | **3.10** | **5.48** |
|  | **14** | **3.86** | **4.53** | **3.80** | **4.45** | **4.00** | **6.73** |
|  | **15** | **3.88** | **4.27** | **4.02** | **3.63** | **4.00** | **4.70** |
|  | **16** | **2.96** | **3.69** | **3.26** | **3.68** | **3.70** | **3.37** |

**Supplementary table 6. Effect of PEG on controlling nutritional status in 6 time periods.**

|  |  | **BT** | **W1** | **W2** | **W3** | **W4** | **MAT** |
| --- | --- | --- | --- | --- | --- | --- | --- |
| **PEG group** | **1** | **1** | **4** | **5** | **4** | **3** | **0** |
|  | **2** | **1** | **2** | **4** | **5** | **4** | **2** |
|  | **3** | **2** | **2** | **0** | **3** | **4** | **1** |
|  | **4** | **1** | **1** | **2** | **3** | **4** | **2** |
|  | **5** | **3** | **4** | **4** | **6** | **6** | **2** |
|  | **6** | **2** | **5** | **5** | **5** | **4** | **4** |
|  | **7** | **9** | **7** | **8** | **7** | **7** | **2** |
|  | **8** | **4** | **3** | **5** | **4** | **4** | **2** |
|  | **9** | **2** | **1** | **1** | **3** | **4** | **1** |
|  | **10** | **1** | **3** | **2** | **5** | **5** | **3** |
|  | **11** | **0** | **0** | **4** | **4** | **3** | **1** |
|  | **12** | **2** | **2** | **5** | **5** | **4** | **1** |
|  | **13** | **0** | **1** | **3** | **3** | **3** | **3** |
|  | **14** | **1** | **5** | **5** | **4** | **4** | **2** |
|  | **15** | **1** | **3** | **3** | **3** | **3** | **4** |
|  | **16** | **3** | **2** | **1** | **3** | **5** | **2** |
|  | **17** | **0** | **3** | **4** | **3** | **3** | **0** |
|  | **18** | **0** | **2** | **3** | **4** | **5** | **3** |
| **Non-PEG group** | **1** | **0** | **3** | **2** | **3** | **4** | **2** |
|  | **2** | **0** | **0** | **3** | **3** | **3** | **1** |
|  | **3** | **2** | **1** | **3** | **5** | **4** | **0** |
|  | **4** | **3** | **0** | **3** | **7** | **4** | **0** |
|  | **5** | **3** | **4** | **5** | **4** | **4** | **3** |
|  | **6** | **1** | **2** | **2** | **2** | **2** | **2** |
|  | **7** | **2** | **4** | **5** | **6** | **5** | **0** |
|  | **8** | **1** | **4** | **3** | **3** | **4** | **0** |
|  | **9** | **1** | **2** | **4** | **4** | **3** | **2** |
|  | **10** | **2** | **1** | **4** | **6** | **5** | **3** |
|  | **11** | **0** | **1** | **3** | **3** | **3** | **2** |
|  | **12** | **2** | **3** | **5** | **5** | **4** | **5** |
|  | **13** | **1** | **3** | **4** | **5** | **4** | **0** |
|  | **14** | **3** | **4** | **4** | **4** | **4** | **0** |
|  | **15** | **1** | **1** | **3** | **4** | **4** | **1** |
|  | **16** | **7** | **2** | **4** | **3** | **3** | **5** |

**Supplementary table 7. The effect of PEG on hemoglobin in 6 time periods.**

|  |  | **BT** | **W1** | **W2** | **W3** | **W4** | **MAT** |
| --- | --- | --- | --- | --- | --- | --- | --- |
| **PEG group** | **1** | **137** | **119** | **109** | **117** | **112** | **140** |
|  | **2** | **117** | **109** | **107** | **93** | **110** | **126** |
|  | **3** | **149** | **140** | **147** | **137** | **134** | **134** |
|  | **4** | **114** | **114** | **115** | **111** | **106** | **120** |
|  | **5** | **122** | **123** | **116** | **112** | **110** | **139** |
|  | **6** | **121** | **115** | **125** | **126** | **120** | **120** |
|  | **7** | **71** | **115** | **116** | **120** | **118** | **122** |
|  | **8** | **110** | **119** | **126** | **123** | **120** | **131** |
|  | **9** | **135** | **142** | **126** | **122** | **101** | **138** |
|  | **10** | **118** | **108** | **111** | **102** | **92** | **123** |
|  | **11** | **125** | **126** | **125** | **125** | **130** | **138** |
|  | **12** | **126** | **127** | **114** | **116** | **116** | **132** |
|  | **13** | **127** | **128** | **118** | **111** | **107** | **102** |
|  | **14** | **134** | **120** | **122** | **122** | **121** | **122** |
|  | **15** | **123** | **121** | **113** | **121** | **114** | **104** |
|  | **16** | **128** | **117** | **123** | **115** | **111** | **103** |
|  | **17** | **133** | **129** | **137** | **136** | **128** | **129** |
|  | **18** | **123** | **120** | **97** | **105** | **103** | **127** |
| **Non-PEG group** | **1** | **118** | **100** | **106** | **99** | **103** | **115** |
|  | **2** | **78** | **108** | **106** | **92** | **91** | **76** |
|  | **3** | **109** | **109** | **102** | **96** | **84** | **118** |
|  | **4** | **123** | **135** | **126** | **118** | **123** | **131** |
|  | **5** | **149** | **131** | **128** | **130** | **131** | **140** |
|  | **6** | **118** | **119** | **124** | **124** | **121** | **124** |
|  | **7** | **108** | **127** | **128** | **101** | **106** | **100** |
|  | **8** | **111** | **120** | **117** | **117** | **105** | **137** |
|  | **9** | **130** | **125** | **116** | **121** | **118** | **128** |
|  | **10** | **122** | **102** | **102** | **102** | **99** | **93** |
|  | **11** | **151** | **148** | **134** | **132** | **128** | **122** |
|  | **12** | **114** | **107** | **96** | **92** | **91** | **107** |
|  | **13** | **133** | **122** | **101** | **94** | **117** | **132** |
|  | **14** | **109** | **112** | **110** | **108** | **90** | **96** |
|  | **15** | **130** | **122** | **118** | **124** | **114** | **134** |
|  | **16** | **113** | **108** | **104** | **97** | **115** | **100** |
